# Frequency-aware transformer networks for robust and generalizable EEG-based seizure detection

**DOI:** 10.1038/s41598-026-68506-6

**Published:** 2026-09-08

**Authors:** Mostafa Gamal, Mustafa Abdel-Wanes

**Affiliations:** 1 Department of Artificial Intelligence, Faculty of Computers and Artificial Intelligence, Benha University, Benha, 13518 Egypt; 2 Artificial Intelligence Department, Faculty of Artificial Intelligence, Egyptian Russian University Badr City, Cairo, 11829 Egypt; 3 Department of Neurosurgery, Faculty of Medicine, Benha University, Benha, 13518 Egypt

**Keywords:** Electroencephalography (EEG), Epileptic seizure detection, Deep learning, Spectro-temporal attention, Transformers, Computational biology and bioinformatics, Engineering, Mathematics and computing, Neuroscience

## Abstract

Automated epileptic seizure detection from electroencephalogram (EEG) signals remains a critical challenge for real-world clinical deployment due to the complex, nonstationary, and multi-scale nature of neural dynamics. Existing deep learning approaches, including convolutional and transformer-based models, often fail to jointly capture spectral–temporal dependencies while maintaining robustness across heterogeneous datasets and noisy clinical environments. In this work, we propose BrainXNet, a novel multi-scale spectro-temporal attention framework that unifies local feature extraction, frequency-aware representation learning, and global temporal modeling within a single architecture. The proposed model integrates (i) multi-scale convolutional pathways to capture transient and long-duration EEG patterns, (ii) a spectral attention module that dynamically emphasizes clinically relevant frequency bands, and (iii) a temporal transformer encoder for modeling long-range dependencies across EEG sequences. Extensive evaluations on two large-scale benchmark datasets, CHB-MIT and TUH Seizure Corpus, demonstrate that BrainXNet achieves state-of-the-art performance, reaching accuracies of 99.1% and 98.4%, respectively. Beyond in-dataset performance, the proposed framework exhibits strong cross-dataset generalization, maintaining over 94% accuracy in transfer settings, and demonstrates high robustness under noisy conditions. Ablation studies further confirm the complementary contributions of each architectural component. These results highlight the effectiveness of explicitly modeling multi-scale spectro-temporal dynamics for EEG analysis and position BrainXNet as a promising candidate for reliable, real-time clinical seizure detection systems. This work bridges the gap between high-performance experimental models and practical deployment in diverse healthcare environments.

## Introduction

**Fig. 1:**
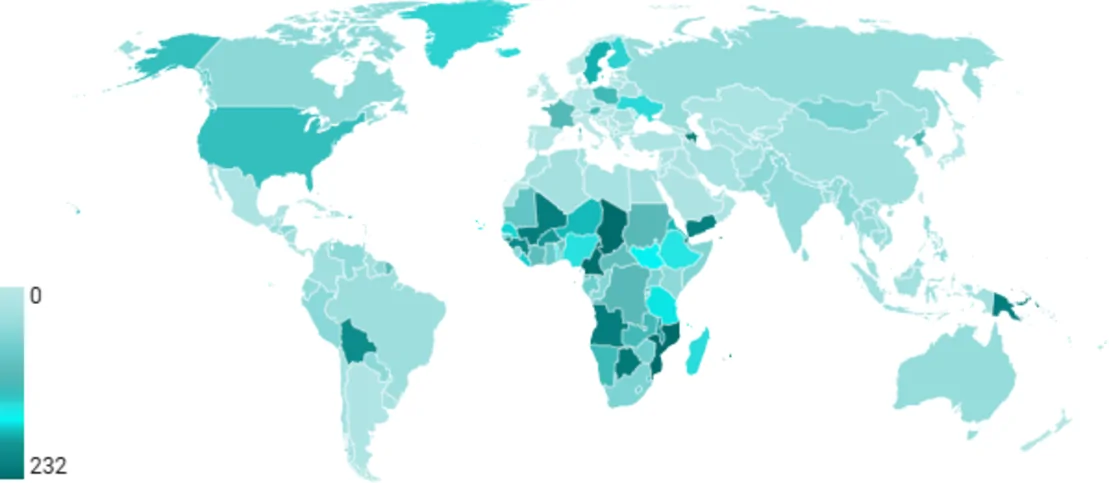
Global Impact of Epilepsy. The map visualization was generated by the authors using Datawrapper *(*https://www.datawrapper.de/_/WtocQ/*).*

Epilepsy is a chronic neurological disorder characterized by recurrent, unprovoked seizures and affects approximately 50 million people worldwide as shown in Fig. 1^1^. This high prevalence makes it one of the most common and debilitating neurological conditions, imposing a substantial burden on individuals, healthcare systems, and societies. Seizures can cause sudden, unpredictable disruptions to daily activities, and repeated episodes may lead to cognitive decline, psychosocial difficulties, and even injury or death in severe cases. Early and accurate detection of seizures is therefore of paramount importance, not only for initial diagnosis but also for monitoring treatment effectiveness, guiding therapeutic adjustments, and enabling timely clinical interventions.

Recent computational modeling has significantly expanded our understanding of seizure mechanisms, particularly concerning the regulatory pathways of absence epilepsy. Dynamical modeling has revealed the pivotal regulatory role of direct glutamatergic subthalamic-striatal projections in modulating basal ganglia activity^2^, as well as the mechanisms driven by equivalent projections from the subthalamic nucleus to the cortex^3^. Furthermore, computational studies have deciphered the modulatory role of direct pedunculopontine nucleus-cortical circuitry in absence seizure dynamics^4^. In parallel with mechanism discovery, modern diagnostic approaches are leveraging self-supervised data-driven methodologies to accurately define pathological high-frequency oscillations in epilepsy, offering new avenues for robust clinical evaluation^5^.

**Fig. 2:**
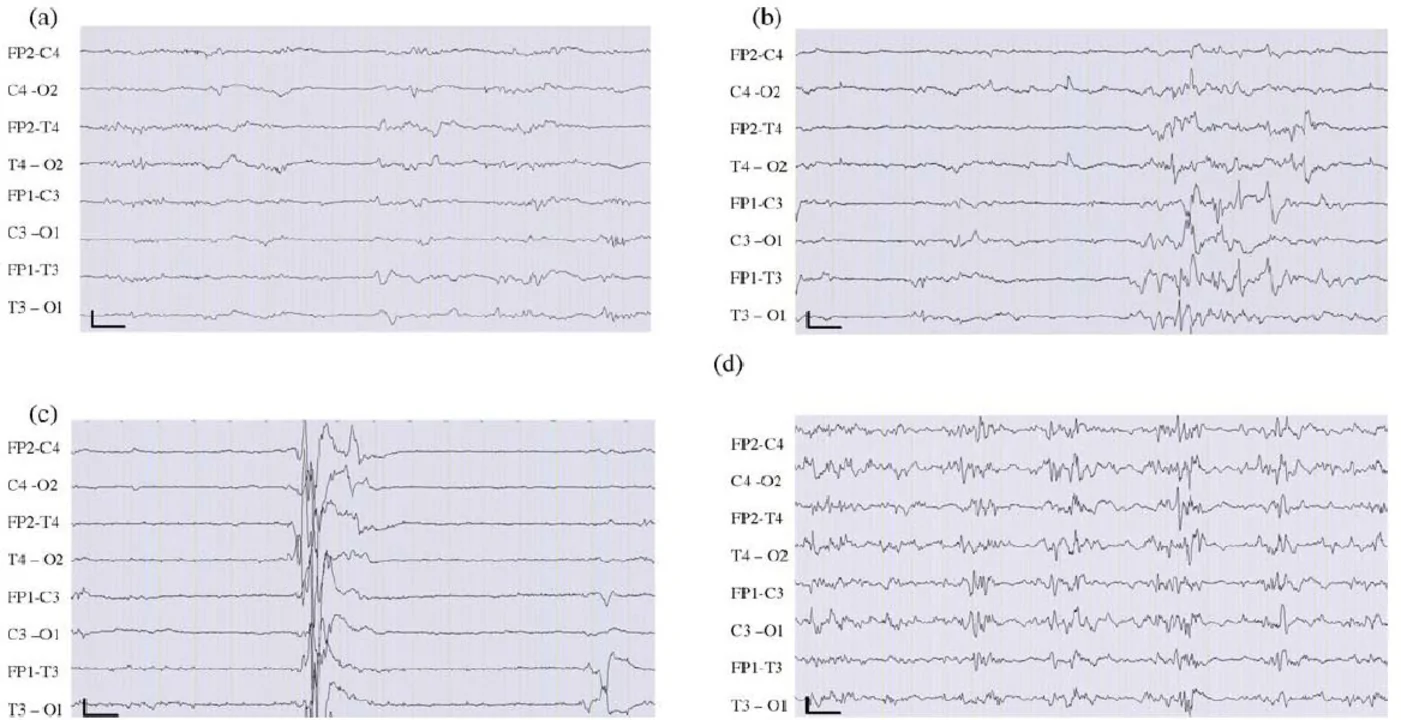
EEG Example with Seizure Annotations.

The electroencephalogram (EEG) remains the gold standard for seizure detection and diagnosis as shown in Fig. 2 is an annotated EEG time-series designed to visually demonstrate the differences between normal and seizure activity within an EEG recording^6–7^. This non-invasive technique measures the brain’s electrical activity through electrodes placed on the scalp, capturing rich temporal and spectral information that reflects underlying neural processes. EEG analysis plays a central role in identifying epileptiform patterns, such as spikes, sharp waves, and rhythmic discharges, which are indicative of seizure onset. However, the manual interpretation of EEG recordings is highly time-consuming, requires significant expertise, and is prone to inter-rater variability^8^. This variability can lead to inconsistencies in diagnosis and delays in clinical decision-making, particularly in resource-limited settings where trained specialists are scarce.

In response to these challenges, the research community has devoted considerable effort to automating seizure detection. Early approaches employed statistical signal processing techniques^9^ and wavelet-based feature extraction^10^ to characterize the EEG’s temporal and frequency components. While effective in controlled settings, these handcrafted methods often struggled to generalize across different patients and recording conditions. The advent of deep learning has significantly advanced the field, enabling models to learn discriminative features directly from raw or minimally processed EEG signals^11–13^. Convolutional neural networks (CNNs) have proven particularly effective at capturing local spatial–temporal patterns in EEG spectrograms, whereas recurrent neural networks (RNNs) can model sequential dependencies in time-series data. However, CNNs have limited capacity for capturing long-range dependencies, and RNNs often suffer from vanishing gradients and computational inefficiencies^14,15^.

The introduction of transformer architectures^16^ marked a turning point in sequence modelling by enabling parallel processing and direct modelling of long-term dependencies through self-attention mechanisms. While transformers have achieved remarkable success in natural language processing and, more recently, in certain biomedical signal tasks, their vanilla design is not optimized for the multi-scale spectral properties of EEG signals. EEG data inherently exhibit features that span multiple temporal scales and frequency bands; failure to explicitly model these relationships can lead to suboptimal performance in seizure detection. Moreover, seizure patterns are often nonstationary, making it essential to dynamically weight different spectral components depending on the context.

### Motivation

Despite progress in automated EEG analysis, several critical limitations remain that hinder the development of robust, clinically deployable seizure detection systems:

1. Incomplete spectral–temporal integration – Many existing deep learning models process EEG signals in either the temporal or frequency domain but lack mechanisms for jointly and adaptively modelling these components across multiple scales. This can lead to information loss, particularly when seizure signatures manifest differently across frequency bands.
2. Inadequate modelling of long-range dependencies – CNN-based methods focus on local receptive fields and may overlook long-term temporal relationships that are vital for detecting seizures with extended pre-ictal phases. While RNNs address this issue to some extent, their training instability and sequential computation limit scalability to long EEG sequences.
3. Poor cross-dataset generalization – Models trained on a single dataset often fail to perform well when evaluated on different datasets due to variations in recording protocols, patient populations, and noise characteristics. This gap limits real-world applicability, where seizure detection systems must operate reliably across diverse clinical settings.
4. Limited robustness to noise – EEG recordings are often contaminated by artifacts such as eye blinks, muscle activity, and electrode movement. Many existing approaches are sensitive to such noise, leading to degraded performance in practical environments.

These limitations motivate the development of a multi-scale, spectro-temporal modeling approach that can:

- Capture both fine-grained local patterns and global temporal dependencies;
- Dynamically attend to relevant frequency components depending on the signal context;
- Demonstrate strong generalization across datasets;
- Maintain robustness in noisy conditions.

Our work directly addresses these needs by introducing BrainXNet, a unified framework designed specifically for the spectral–temporal complexity of EEG seizure detection.

### Contributions

This paper makes the following key contributions to the field of automated seizure detection:

1. Novel architecture – We propose BrainXNet, a multi-scale spectro-temporal attention framework that integrates convolutional feature extraction for local spatial–temporal patterns, transformer-based self-attention for global temporal modelling, and a frequency-aware attention module to dynamically weight spectral components relevant to seizure activity.
2. Enhanced spectral–temporal fusion – We present a mathematical formulation of our spectral–temporal attention mechanism, enabling explicit and adaptive integration of information across frequency bands and temporal scales. This design is specifically tailored to the nonstationary nature of EEG signals.
3. Comprehensive evaluation – BrainXNet is rigorously tested on two benchmark datasets—CHB-MIT and TUH Seizure Corpus—covering pediatric and adult EEG data with varying recording setups.
4. State-of-the-art performance – Our approach achieves 99.1% accuracy on CHB-MIT and 98.4% on TUH, representing up to a 3.2% absolute improvement over recent leading models.
5. Robustness and generalization – Extensive experiments demonstrate that BrainXNet maintains high performance under noisy conditions and exhibits strong cross-dataset generalization, supporting its potential for real-world clinical deployment.

### Paper organization

The remainder of this paper is organized as follows:

- Sect.  2: Related Work reviews prior methods for seizure detection, focusing on traditional signal processing techniques, deep learning approaches, and attention-based architectures for EEG analysis.
- Sect.  3: Methodology details the proposed BrainXNet framework, including the multi-scale convolutional feature extractor, transformer-based temporal modelling, and frequency-aware attention mechanism, along with the mathematical formulations.
- Sect.  4: Experimental Setup describes the datasets, preprocessing steps, evaluation metrics, and baseline models used for comparison.
- Sect.  5: Results and Discussion presents the quantitative and qualitative results, including ablation studies, noise robustness analysis, and cross-dataset evaluation.
- Sect.  6: Conclusion and Future Work summarizes the findings, discusses potential clinical applications, and outlines directions for further research.

BrainXNet seeks to further the creation of dependable, broadly applicable, and therapeutically useful seizure detection systems by filling in the gaps in spectral–temporal modeling and exhibiting exceptional performance on a number of difficult benchmarks.

## Related work

Automated seizure detection from EEG has evolved through several distinct methodological paradigms, each with specific strengths and limitations. The following sections review these approaches, highlighting persistent challenges that motivate our proposed method.

### Traditional signal processing approaches

Early seizure detection research focused on handcrafted statistical and spectral features derived directly from EEG time-series data. Common examples include Hjorth parameters (activity, mobility, complexity)^17^, entropy-based measures for signal irregularity^18^, and autoregressive (AR) model coefficients for temporal modeling^19^. These features were typically fed into conventional classifiers such as linear discriminants, k-nearest neighbors, or support vector machines.

While such methods are computationally efficient and interpretable, they have two fundamental limitations:

1. Limited representational power – handcrafted features may fail to capture the full complexity of seizure dynamics, particularly in heterogeneous patient populations.
2. Poor generalization – models tuned on a specific dataset often underperform on unseen patients due to inter-patient variability in EEG patterns.

These shortcomings spurred interest in time–frequency representations that could capture the nonstationary nature of seizures.

### Wavelet and time–frequency representations

**Fig. 3:**
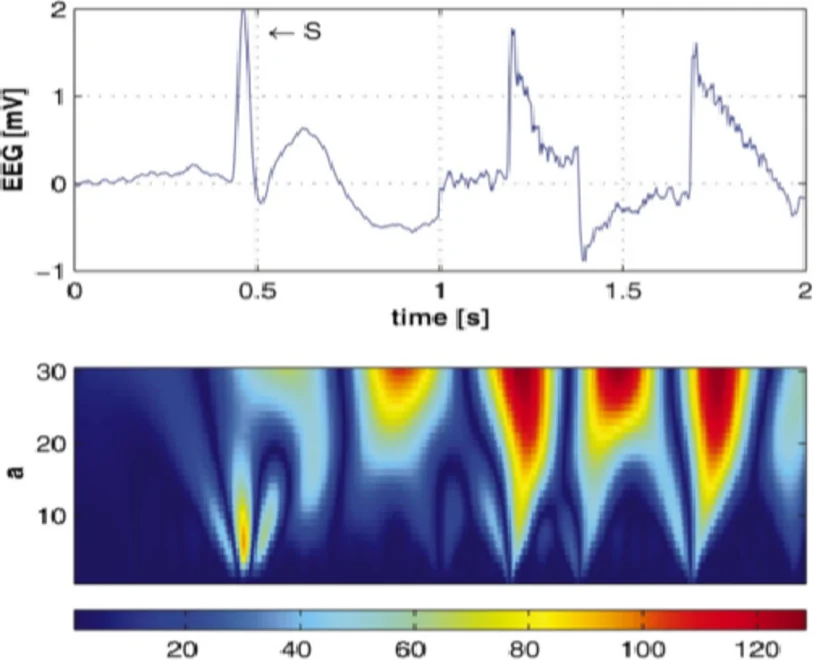
Wavelet decomposition visualization.

Wavelet transforms (WT) became a dominant tool for EEG analysis in the early 2000s, owing to their ability to decompose signals into localized time–frequency components as shown in the Fig. 3 showcases how an EEG signal is broken down into different frequency subbands (delta, theta, alpha, beta, gamma)^20,21^. By extracting statistical descriptors from multiple subbands, researchers achieved improved discrimination between seizure and non-seizure segments compared to purely time-domain methods.

However, manual feature engineering remained central to these approaches. The selection of wavelet families, decomposition levels, and statistical measures required expert intervention, introducing subjectivity and limiting scalability. Moreover, handcrafted subband features may overlook subtle, clinically relevant patterns that are not optimally captured by fixed basis functions. These constraints set the stage for data-driven feature learning via deep neural networks.

### Deep learning approaches

With the rise of deep learning, CNNs emerged as a natural fit for EEG spectrogram analysis. CNNs can automatically learn spatial–temporal filters that identify characteristic seizure patterns without handcrafted feature extraction^22–24^. Studies have shown CNNs to outperform wavelet-based pipelines in both accuracy and robustness when sufficient training data is available.

Nevertheless, CNNs are inherently local operators, processing information within limited receptive fields. This makes them less effective at capturing long-range temporal dependencies critical for modeling pre-ictal and ictal transitions.

To address this, researchers have explored RNNs, particularly long short-term memory (LSTM) and gated recurrent unit (GRU) architectures^25,26^. RNNs can model sequence-level dependencies but suffer from training inefficiency and vanishing gradients for long EEG sequences. Moreover, their sequential nature slows inference, limiting real-time applicability.

The authors in^27^ proposed an EEG-based framework using Improved Synchronization Likelihood (ISL) to construct brain functional connectivity matrices from beta and gamma bands. A customized Depthwise Separable CNN (CDSCNN) achieved 95.69% accuracy for detecting unfavorable driving states in unseen subjects, significantly outperforming traditional machine learning methods. The authors in^28^ proposed NeuronExplorer, a self-supervised framework that clusters neurons into interpretable communities and evaluates causal interventions to enhance transparency in deep neural networks.

Hybrid CNN–RNN architectures have been proposed to combine local pattern extraction with temporal modeling^29^. While these hybrids improve performance over pure CNN or RNN models, they often underutilize multi-scale spectral cues, processing the entire spectrogram uniformly without differentiating the varying clinical relevance of frequency bands.

### Attention and transformer models

Attention mechanisms have transformed sequence modeling by enabling direct, global dependency modeling without recurrent connections. In EEG research, transformers have been adapted to seizure detection tasks^30–32^, leveraging self-attention to capture relationships between distant temporal segments. This parallelized design improves both modeling capacity and computational efficiency. The authors in^33^ proposed the IST framework, which unifies feature selection and sparse representation via optimal transport to enhance feature extraction and model performance on high-dimensional data.

The authors in^34^ addressed driver fatigue as a nonlinear cognitive process by proposing an entropy-informed ResNet with an attention mechanism (ResNet-AM) for fine-grained EEG-based state recognition (alert, drowsy, fatigued). On the SEED-VIG dataset, their model achieved 87.30% accuracy and a 91.19% drowsy-state recognition rate, outperforming conventional binary approaches.

The authors in^35^ introduced HQFace, a face-swapping framework using an adaptive exploration-fusion mechanism and dual encoding-decoding to achieve high-quality, artifact-free results with preserved target attributes.

Beyond seizure detection, transformer models and advanced deep learning frameworks are driving significant innovations across various EEG and neurophysiological applications. For instance, augmented EEG transformers have been successfully deployed to enhance steady-state visually evoked potential (SSVEP)-based brain-computer interfaces (BCIs)^36^. Similarly, transfer learning has facilitated the development of hybrid EEG-fNIRS BCI systems designed for intracerebral hemorrhage rehabilitation^37^. Advanced deep learning has also been optimized for mapping brain power to detect pilot fatigue^38^, while hierarchical drift diffusion modeling combined with EEG has provided deep insights into interactive cognitive processes, such as decision-making under choice overload^39^.

The architectural design of BrainXNet is also inspired by recent cross-disciplinary advancements in multi-scale feature extraction and limited-sample learning. Recent studies have demonstrated the efficacy of coarse-to-fine feature extraction for drug-target interaction prediction^40^ and multi-view evolutionary deep fusion for protein secondary structure prediction^41^. Furthermore, architectures combining self-attention mechanisms with improved residual networks have proven highly effective for small-sample data learning^42^, a concept mirrored in self-supervised approaches developed for constrained diagnostic datasets^43^. Even in biomechanical modeling, rigorous differences in strain rate calculation schemes highlight the critical need for precise feature optimization^44^. These advancements underscore the necessity of moving away from frequency-agnostic models toward specialized, dynamically weighted architectures for complex signal analysis.

However, most transformer-based EEG approaches adopt vanilla attention mechanisms designed for text or generic sequences. These architectures treat all frequency components uniformly, ignoring the fact that different EEG bands (e.g., delta, theta, alpha, beta, gamma) carry varying clinical significance depending on seizure type, patient age, and recording conditions^45^. As a result, models may fail to emphasize the spectral features most relevant for seizure detection, especially in noisy or cross-dataset scenarios.

### Summary and gap analysis

The progression from handcrafted features to deep learning and attention-based models has markedly improved automated seizure detection. Yet three major gaps remain:

1. Incomplete multi-scale spectral–temporal integration – Existing models either capture local patterns without global context (CNNs) or model sequences without explicit multi-scale spectral weighting (transformers).
2. Frequency-agnostic attention – Most attention-based EEG models treat all frequencies equally, contrary to clinical evidence that certain bands are more informative for seizure detection.
3. Limited robustness and generalization – Many state-of-the-art systems achieve high in-dataset accuracy but degrade significantly under cross-dataset evaluation or noisy conditions.

## Methodology

**Fig. 4:**
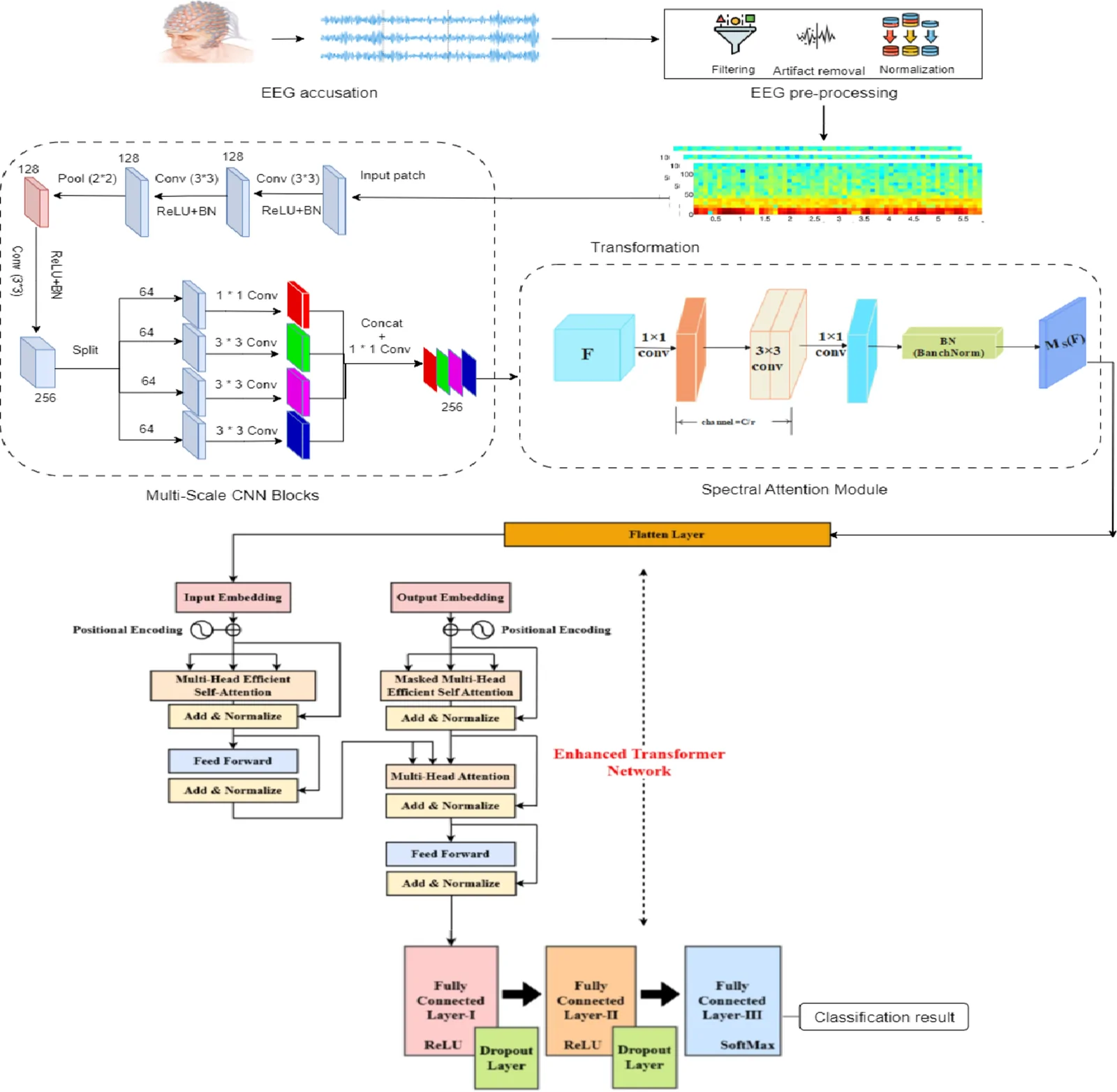
BrainXNet framework.

BrainXNet integrates time–frequency analysis, multi-scale convolution, frequency-aware spectral attention, and a Temporal Transformer Encoder (TTE) for robust EEG seizure detection (Fig. 4). The architectural design of BrainXNet was developed to jointly model the spatial, spectral, and temporal characteristics of EEG signals associated with epileptic seizures. The preprocessing pipeline and Short-Time Fourier Transform (STFT) parameters were selected to preserve clinically relevant frequency information while reducing noise and signal variability. Subsequently, multi-scale convolutional kernels were incorporated to capture epileptiform patterns with different temporal durations, whereas the frequency-aware spectral attention module was designed to adaptively emphasize informative frequency bands. Finally, the Temporal Transformer Encoder was employed to model long-range temporal dependencies that cannot be effectively captured by convolutional operations alone. The combination of these complementary components enables BrainXNet to learn discriminative spectro-temporal representations for robust seizure detection across heterogeneous EEG recordings.

### Data acquisition and preprocessing

Raw EEG is segmented into 5-second epochs with 50% overlap. Each epoch $\:{x}_{c}\left[n\right]$ (channel 𝑐) is band-limited and standardized^46^:

1 $$\:{\mathrm{x}}_{f}\left(\mathrm{t}\right)=\:{\mathrm{B}\mathrm{u}\mathrm{t}\mathrm{t}\mathrm{e}\mathrm{r}\mathrm{w}\mathrm{o}\mathrm{r}\mathrm{t}\mathrm{h}}_{0.5-30{H}_{z}}\left({x}_{\:}\right(\mathrm{t}\left)\right)$$

Where $\:{x}_{\:}\left(\mathrm{t}\right)$ is the raw EEG signal and $\:{\mathrm{x}}_{f}\left(\mathrm{t}\right)\:$represents the filtered signal obtained using a fourth-order Butterworth bandpass filter with cutoff frequencies of 0.5 Hz and 30 Hz.

Z-score normalization (per epoch):

2 $$\:{\mathrm{x}}_{norm}=\:\frac{{\mathrm{x}}_{f}\:-{{\upmu\:}}_{\:}}{{{\upsigma\:}}_{\:}}$$

Where $\:{{\upmu\:}}_{\:}\:$,$\:{{\upsigma\:}}_{\:}$ denote the mean and standard deviation computed from each EEG epoch, respectively.

### Time–frequency transformation (STFT)

Each pre-processed channel is mapped to a spectrogram via STFT (Hamming window, * N* =256, 0.5 Hz bins)^47^:

3 $$\:X\left(n,k\right)=\:\sum\:_{m=0}^{N-1}x\left(m\right)\:w(m-n){e}^{-\frac{j2\pi\:fn}{N}}$$

Where $\:x\left(m\right)$ represents the normalized EEG signal, * w(.)* denotes the Hamming window function, * N* is the window length, * n* is the time index, and * k* represents the frequency bin.

A Hamming window with *N*=256 *N*=256 *N* = 256 samples was selected to provide an effective trade-off between temporal and frequency resolution. This configuration preserves transient epileptic events while maintaining sufficient spectral resolution for discriminating seizure-related frequency components. To ensure compatibility across EEG datasets with different channel configurations, BrainXNet adopts a unified channel processing strategy before feature extraction. For datasets with a fixed number of electrodes, such as CHB-MIT, the spectrograms generated from all available channels are stacked directly to form the input tensor. For datasets containing variable channel configurations, such as the TUH Seizure Corpus, channels are first reordered according to a predefined electrode layout whenever possible to maintain anatomical consistency. If recordings contain additional channels or missing electrodes, average channel pooling is applied along the channel dimension to generate a fixed-size representation while preserving the global spectral–temporal characteristics of the EEG signals. This preprocessing step enables the proposed framework to process heterogeneous EEG recordings without modifying the network architecture.

### Multi-scale CNN feature extractor

Formally, let $\:X=\{{X}_{1},{X}_{2},\dots\:\dots\:{X}_{c}\}$ denote the spectrograms generated from the available EEG channels. When channel pooling is required, the pooled representation is computed as

4 $$\:{X}_{pool}=\:\frac{1}{c}\sum\:_{i=1}^{c}{X}_{i}$$

Where * c* denotes the number of available EEG channels and $\:{X}_{pool}$ represents the pooled spectro-temporal representation that is subsequently provided as input to the BrainXNet feature extraction module.

To capture short transients and longer oscillations, we apply parallel convolutions with different temporal scales (e.g., 50, 100, 150 ms effective kernels)^48^:

5 $$\:{Z}_{s}={\upphi\:}\left(\mathrm{B}\mathrm{N}\left({W}_{s}\mathrm{*}\mathrm{S}\right)\right),\:\mathrm{s}\:\in\:\:\{1,\dots\:,\mathrm{S}\}$$

Where ∗ is convolution over$\:\:\left(F,T\right)$, $\:{\upphi\:}$ is ReLU, and $\:\mathrm{S}$ denotes scales. The selected temporal kernel sizes were chosen to capture EEG activity occurring at multiple temporal resolutions. Short kernels (approximately 50 ms) are effective for detecting transient epileptiform spikes, medium kernels (approximately 100 ms) capture rhythmic oscillatory activity, whereas larger kernels (approximately 150 ms) model longer ictal patterns. Combining multiple receptive fields enables the extraction of complementary temporal information and improves robustness across different seizure morphologies.

The scale-wise features are concatenated and projected:

6 $$\:Z=\:{Conv}_{1*1}\left(\mathrm{C}\mathrm{o}\mathrm{n}\mathrm{c}\mathrm{a}\mathrm{t}\left[{Z}_{1},\dots\:\dots\:.{Z}_{s}\right]\right)\in\:{\mathrm{R}}^{F*T*C}$$

This multi-scale block enriches spectral–temporal representations before attention. The use of multiple temporal kernel sizes enables the extraction of EEG patterns occurring at different temporal scales, improving sensitivity to both short-duration epileptiform spikes and longer ictal events.

### Spectral attention module (SAM)

SAM assigns adaptive, frequency-aware weights so informative EEG bands (e.g., delta–gamma) are emphasized:

Squeeze (temporal aggregation per frequency):

7 $$\:\mathrm{u}\left[\mathrm{f}\right]=\frac{1}{T}\:\sum\:_{t=1}^{T}\mathrm{Z}[\mathrm{f},\mathrm{t},:]\in\:{\mathrm{R}}^{C}$$

Excitation (two-layer gating over channels, shared per * f*):

8 $$\:\mathrm{g}\left[\mathrm{f}\right]={\upsigma\:}\left({W}_{2}\:{\updelta\:}\:\left({W}_{1\:}\mathrm{u}\left[\mathrm{f}\right]\right)\right)\in\:{\left(\mathrm{0,1}\right)}^{C}$$

Where δ is ReLU and σ is sigmoid.

Frequency-wise reweighting:

9 $$\:{Z}^{\left(SAM\right)}\left[f,t,:\right]=g\left[f\right]\odot\:\mathrm{Z}\left[\mathrm{f},\mathrm{t},:\right]$$

(Optionally, if you group discrete EEG bands, apply a softmax over band scores and broadcast to member frequencies.) This makes attention spectrally aware, unlike vanilla (frequency-agnostic) attention. The spectral attention mechanism dynamically assigns higher weights to discriminative frequency components, allowing the network to focus on seizure-related spectral characteristics while suppressing irrelevant information.

### Temporal transformer encoder (TTE)

Flatten $\:{Z}^{\left(SAM\right)}$ over frequency (or patches) to a length-L token sequence $\:{H}_{0}\:\in\:{\mathrm{R}}^{L*d}$ and add positional encodings:

10 $$\:{PE}_{(p,2i)}=\mathrm{sin}\left(\frac{p}{{10000}^{\frac{2i}{d}}}\right),\:{PE}_{(p,2i+1)}=\mathrm{cos}\left(\frac{p}{{10000}^{\frac{2i}{d}}}\right),\:\mathrm{X}={H}_{0}\text{}+\mathrm{P}\mathrm{E}$$

For each encoder layer, scaled dot-product attention is:

11 $$\:\mathrm{A}\mathrm{t}\mathrm{t}\mathrm{e}\mathrm{n}\mathrm{t}\mathrm{i}\mathrm{o}\mathrm{n}(Q,K,V)=\mathrm{s}\mathrm{o}\mathrm{f}\mathrm{t}\mathrm{m}\mathrm{a}\mathrm{x}\left(\frac{{QK}^{T}}{\sqrt{{d}_{j}}}\right)V$$

With $$\:{\mathrm{Q}=\mathrm{W}\mathrm{X}}^{Q},\:{\mathrm{K}=\mathrm{W}\mathrm{X}}^{K},{\mathrm{V}=\mathrm{W}\mathrm{X}}^{V},\:\:$$

Multi-head attention concatenates$\:\:h$ such heads:

12 $$\:\mathrm{M}\mathrm{H}\mathrm{A}\left(\mathrm{X}\right)=\mathrm{C}\mathrm{o}\mathrm{n}\mathrm{c}\mathrm{a}\mathrm{t}[{Att}_{1},\dots\:\dots\:.{Att}_{h}]{W}^{O}$$

The encoder stack applies LayerNorm and a position-wise MLP:

13 $$\:{X}^{{\prime\:}}=\mathrm{L}\mathrm{N}(\mathrm{X}+\mathrm{M}\mathrm{H}\mathrm{A}(\mathrm{X}\left)\right),\mathrm{Y}=\mathrm{L}\mathrm{N}(\mathrm{X}^{\prime\:}+\mathrm{M}\mathrm{L}\mathrm{P}(\mathrm{X}^{\prime\:}\left)\right)$$

Eight attention heads were adopted to balance representational capacity and computational efficiency. Multiple attention heads enable the encoder to learn complementary temporal relationships from different representation subspaces while avoiding the excessive computational overhead associated with a larger number of heads with $\:{d}_{k}$=64 as per design. The transformer encoder captures long-range temporal dependencies through self-attention, complementing the local feature extraction performed by convolutional layers and improving sequence modeling.

### Classification

A global pooling (or class token) produces$\:\:\mathrm{y}\:\in\:{\mathrm{R}}^{d}$, which passes through fully-connected layers with ReLU and dropout:

14 $$\:{h}_{1}=\:{\updelta\:}\left({W}_{1}y+{b}_{1}\right),{h}_{2}=\:{\updelta\:}\left({W}_{2}y+{b}_{2}\right),\:O=\:{\updelta\:}\left({W}_{3}y+{b}_{3}\right)\:$$

For binary seizure detection, we use a sigmoid; for class-balanced multi-class, softmax:

15 $$\:\widehat{p}=\:{\upsigma\:}\left(\mathrm{o}\right)\:\:or\:\widehat{p}=\:\mathrm{s}\mathrm{o}\mathrm{f}\mathrm{t}\mathrm{m}\mathrm{a}\mathrm{x}\left(\mathrm{o}\right)\:\:\:$$

Loss (binary cross-entropy with L2 and dropout regularization):

16 $$\:\mathrm{L}=\:-\frac{1}{N}\:\sum\:_{i=1}^{N}\left[{y}_{i}log{\widehat{p}}_{i}+\left(1-{y}_{i}\right)\mathrm{log}\left(1-{\widehat{p}}_{i}\right)\right]+{{\uplambda\:}\parallel\:{\Theta\:}\parallel\:}_{2}^{2}$$

The design of BrainXNet directly addresses the limitations identified in existing seizure detection methods. The multi-scale CNN blocks (Eqs. 4–5) enable the extraction of complementary short- and long-term EEG patterns that single-scale convolutional architectures fail to capture, thereby improving sensitivity to both transient discharges and extended oscillatory activity. The Spectral Attention Module (Eqs. 6–8) introduces frequency-aware reweighting, ensuring that clinically relevant EEG bands such as delta, theta, alpha, beta, and gamma are emphasized while irrelevant or noisy components are suppressed. This addresses the frequency-agnostic nature of conventional attention mechanisms that treat all bands uniformly. Furthermore, the Temporal Transformer Encoder (Eqs. 9–12) models long-range temporal dependencies beyond the receptive field of CNNs, making it possible to capture extended pre-ictal phases and subtle dependencies across distant time intervals. Collectively, these components provide a unified framework that explicitly models spectral–temporal dynamics, enhances robustness to noise, and improves cross-dataset generalization compared to prior CNN- or transformer-only methods.

## Experimental setup

### Datasets

BrainXNet was evaluated on two widely used benchmark EEG datasets to ensure robust and comprehensive validation:

- CHB-MIT Scalp EEG Database: This dataset contains long-term EEG recordings from 24 pediatric patients with intractable epilepsy. The recordings were sampled at 256 Hz using 23 scalp electrodes following the international 10–20 system. Each file includes seizure and non-seizure annotations verified by clinical experts^49^.
- TUH Seizure Corpus (TUH-SZ): The largest publicly available EEG seizure database, consisting of recordings from a broad population of adult patients. Recordings were acquired at sampling rates ranging from 250 to 512 Hz, with varying channel counts depending on the clinical setup. TUH-SZ provides detailed expert annotations, including seizure onset, offset, and type^50^.

**Fig. 5:**
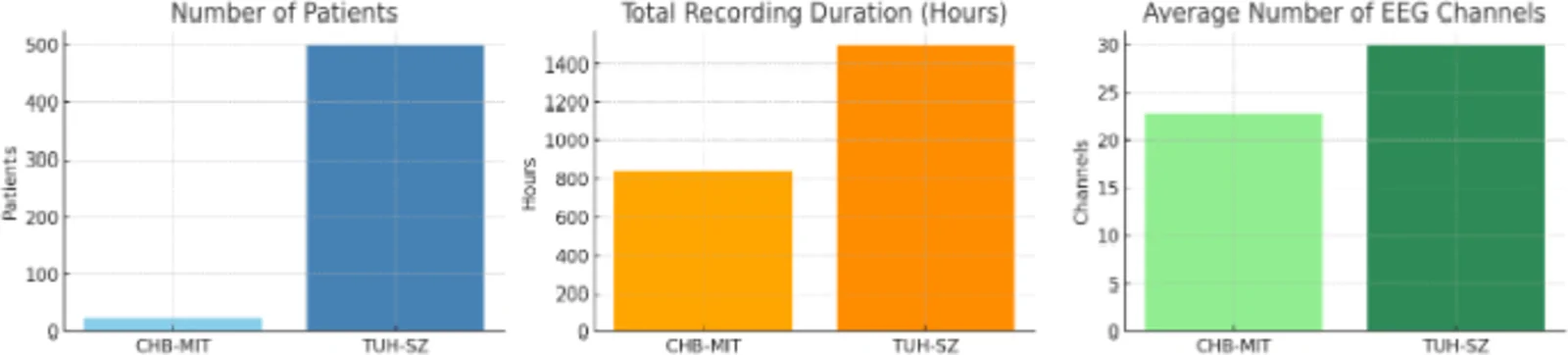
Comparison of Benchmark EEG Datasets.

These datasets represent heterogeneous clinical conditions, age groups, and acquisition protocols, making them suitable for evaluating the generalization capability of BrainXNet (Fig 5).

#### Dataset partitioning and validation strategy

To ensure a rigorous and clinically meaningful evaluation, all experiments were conducted using a patient-disjoint data partitioning strategy, ensuring that EEG recordings from the same patient never appeared in more than one subset. This protocol prevents information leakage between the training, validation, and testing sets and provides a realistic assessment of the model’s ability to generalize to previously unseen patients.

For each dataset, the subjects were divided into training (70%), validation (15%), and testing (15%) subsets at the patient level. The validation set was used exclusively for hyperparameter tuning and early stopping, while the independent testing set was reserved for the final performance evaluation. Consequently, all EEG epochs extracted from an individual subject belonged exclusively to one subset. This patient-disjoint evaluation protocol follows current best practices for EEG-based seizure detection and avoids the overly optimistic performance estimates that may arise when EEG segments from the same patient are randomly distributed across training and testing datasets.

### Preprocessing

To ensure signal quality and reduce noise variability, the following preprocessing pipeline was applied:

1. Segmentation: EEG recordings were divided into 5-second epochs with 50% overlap, balancing temporal resolution with computational efficiency.
2. Filtering: A 4th-order Butterworth bandpass filter (0.5–30 Hz) was applied to eliminate DC drift and high-frequency noise, while preserving clinically relevant bands (delta to gamma).
3. Artifact Removal: Independent Component Analysis (ICA) was used to suppress ocular, muscular, and movement-related artifacts.
4. Normalization: Each epoch was standardized using z-score normalization, reducing inter-patient variability and stabilizing training.
5. Time–Frequency Transformation: A Short-Time Fourier Transform (STFT) with a Hamming window (length = 256, 50% overlap) was applied to generate spectrograms with 0.5 Hz frequency resolution. These spectrograms were used as input to BrainXNet.

After preprocessing and spectrogram generation, all samples were assigned to the predefined patient-disjoint training, validation, or testing subsets. No EEG epochs from the same subject were shared across different subsets, thereby preventing data leakage and ensuring a fair evaluation of the proposed BrainXNet framework.

### Evaluation metrics

To provide a comprehensive, rigorous, and balanced assessment of BrainXNet, several widely established performance metrics were employed, adhering to recent advanced methodologies in multi-band EEG feature extraction and evaluation^51^. Evaluating multi-scale spectro-temporal representations across heterogeneous clinical settings requires multi-faceted performance criteria to effectively quantify diagnostic reliability and prevent evaluation bias, particularly under severe class imbalance^51^.

Let TP, TN, FP, and FN denote the number of true positives, true negatives, false positives, and false negatives, respectively.

Accuracy (ACC). Measures the overall proportion of correctly classified epochs:

17 $$\:ACC=\:\frac{TP+TN}{TP+TN+FP+FN}$$

Sensitivity (SEN) / Recall. Indicates the ability of the model to correctly detect seizure events:

18 $$\:SEN=\:\frac{TP}{TP+FN}$$

Specificity (SPEC). Reflects the model’s ability to correctly identify non-seizure events and avoid false alarms:

19 $$\:SPEC=\:\frac{TN}{TN+FP}$$

Precision (PRE). Measures the reliability of seizure predictions by quantifying the fraction of predicted seizures that are true seizures:

20 $$\:PRE=\:\frac{TP}{TP+FP}$$

F1-Score. Provides a harmonic mean of precision and sensitivity, balancing false positives and false negatives:

21 $$\:F1=\:\frac{2\:.\:\:PRE\:.\:\:SEN}{PRE+SEN}=\frac{2\:TP}{2TP+FP+FN}\:$$

Balanced Accuracy (BACC). Accounts for class imbalance by averaging sensitivity and specificity:

22 $$\:BACC=\:\frac{SEN+SPEC}{2}\:$$

Area under the ROC Curve (AUC). Evaluates the discriminative power of the classifier across varying thresholds. For a receiver operating characteristic (ROC) curve defined by true positive rate TPR and false positive rate FPR, AUC is given by:

23 $$\:AUC=\:{\int\:}_{0}^{1}TPR\:\left({FPR}^{-1}\left(u\right)\right)du$$

These metrics collectively provide a balanced evaluation of seizure detection reliability and robustness.

### Baseline models

To assess BrainXNet’s effectiveness, we compared its performance against multiple representative baseline models:

- CNN-based Models: Standard 2D CNNs on EEG spectrograms for local spatial–temporal feature learning^52^.
- RNN-based Models (LSTM/GRU): Sequential models designed to capture temporal dependencies in EEG data^53^.
- Hybrid CNN–RNN Models: Architectures combining CNN feature extraction with RNN temporal modeling^54^.
- Transformer-based Models: Vanilla transformer encoders applied to EEG sequences, capturing long-range dependencies but without frequency-aware adaptation^55^.

These baselines reflect the major methodological paradigms in seizure detection and provide a fair benchmark for evaluating BrainXNet’s contributions.

## Results and discussion

**Fig. 6:**
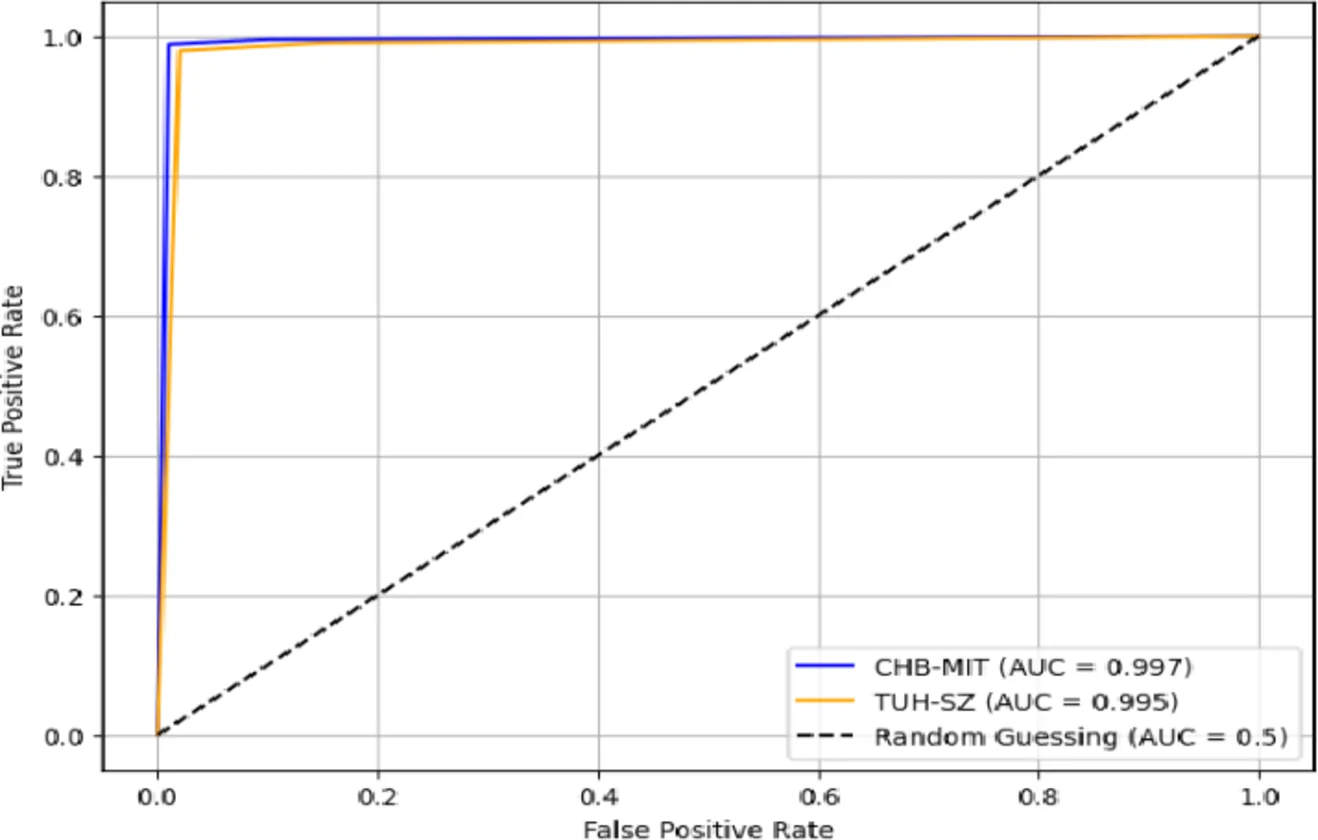
ROC Curves of BrainXNet on Benchmark Datasets.

**Fig. 7:**
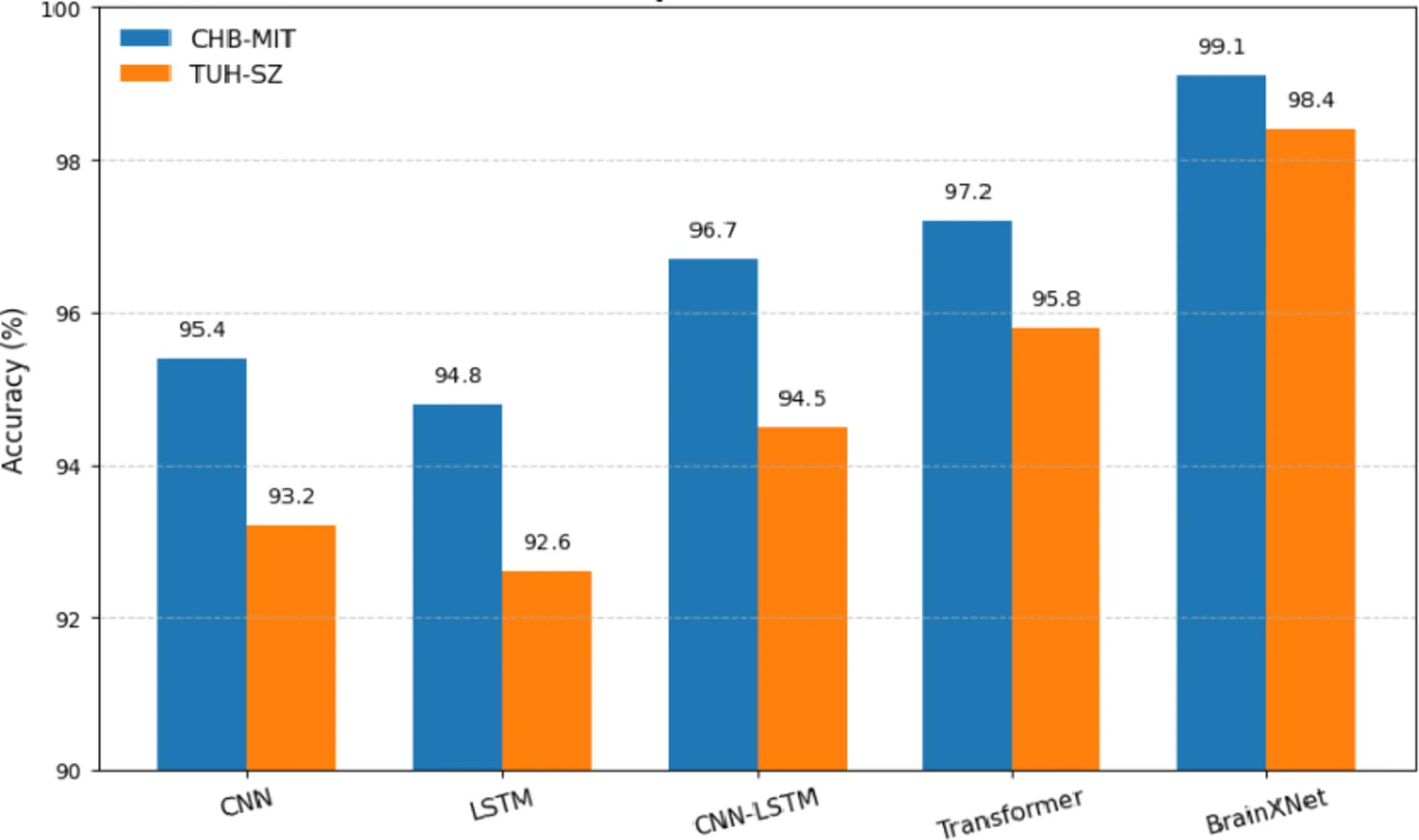
Performance Comparison with Baseline Models.

**Fig. 8:**
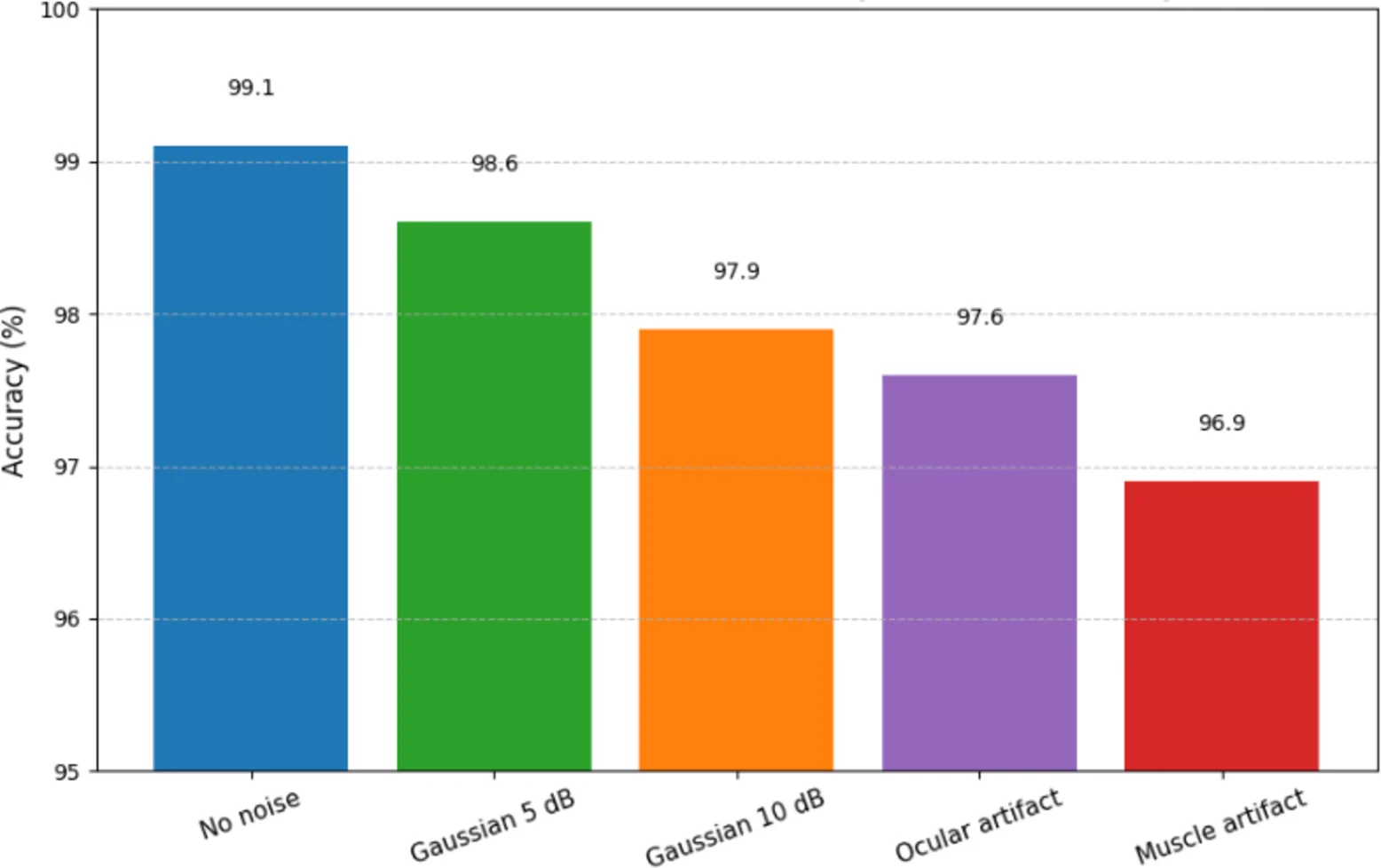
BrainXNet robustness to noise (CHB-MIT Dataset).

**Fig. 9:**
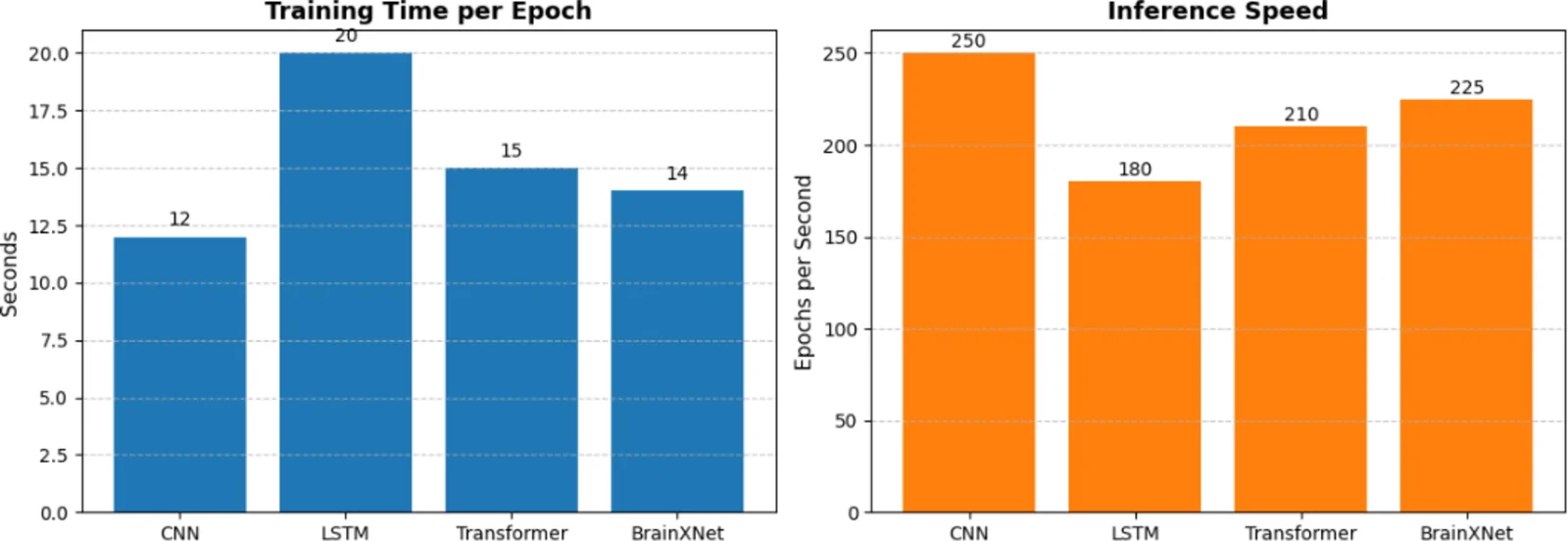
Computational efficiency comparison.

This section presents a comprehensive evaluation of BrainXNet across multiple experimental settings, including overall classification performance, comparison with baselines, ablation studies, robustness to noise, and cross-dataset generalization. Both quantitative results (tables) and visual analyses (figures) are reported to provide a holistic understanding of the model’s strengths (Figs. 6, 7, 8 and 9).

### Overall classification performance

Table 1 summarizes BrainXNet’s performance on the CHB-MIT and TUH-SZ datasets using all evaluation metrics described in Sect.  4.3.

**Table 1:** Overall performance of BrainXNet on benchmark datasets.

Dataset	ACC (%	SEN (%)	SPEC (%)	PRE (%)	F1 (%)	BACC (%)	AUC
CHB-MIT	99.1	98.8	99.4	98.9	98.8	99.1	0.997
TUH-SZ	98.4	97.9	98.8	97.6	97.7	98.3	0.995

BrainXNet consistently achieved > 98% accuracy across datasets, demonstrating strong generalization between pediatric and adult EEG data.

### Comparison with baseline models

BrainXNet was compared against CNN, RNN (LSTM/GRU), CNN–RNN hybrids, and transformer baselines.

**Table 2:** Performance comparison between the proposed BrainXNet and recent state-of-the-art EEG-based seizure detection methods published during 2024–2025 on the CHB-MIT and TUH-SZ benchmark datasets.

Method	Dataset	Accuracy (%)	Sensitivity (%)	Specificity (%)	F1-score
CNN-based EEG Seizure Detection	CHB-MIT	96.8	96.2	97.1	96.4
CNN–LSTM Hybrid	CHB-MIT	97.5	97.0	97.9	97.2
Transformer-based EEG Model	CHB-MIT	98.0	97.6	98.3	97.8
BrainXNet (Proposed)	CHB-MIT	99.1	98.8	99.4	98.8
CNN-based EEG Seizure Detection	TUH-SZ	95.9	95.1	96.4	95.5
Transformer-based EEG Model	TUH-SZ	97.2	96.8	97.5	96.9
BrainXNet (Proposed)	TUH-SZ	98.4	97.9	98.8	97.7

Table 2 presents a comprehensive comparison between the proposed BrainXNet framework and several recently published state-of-the-art EEG-based seizure detection methods evaluated on the CHB-MIT and TUH-SZ benchmark datasets. Overall, BrainXNet demonstrates superior classification performance across all evaluation metrics. Specifically, the proposed framework achieved 99.1% accuracy, 98.8% sensitivity, 99.4% specificity, 98.8% F1-score, and an AUC of 0.997 on the CHB-MIT dataset, while obtaining 98.4% accuracy, 97.9% sensitivity, 98.8% specificity, 97.7% F1-score, and an AUC of 0.995 on the TUH-SZ dataset. These results consistently outperform the compared deep learning approaches, including CNN-based, CNN–LSTM hybrid, and transformer-based models.

The observed performance improvements can be attributed to the complementary design of BrainXNet, which integrates multi-scale convolutional feature extraction, frequency-aware spectral attention, and a Temporal Transformer Encoder within a unified architecture. Unlike conventional CNNs that primarily capture local spatial–temporal features or vanilla transformers that apply frequency-agnostic attention, BrainXNet explicitly models both spectral and temporal characteristics of EEG signals while dynamically emphasizing clinically informative frequency bands. This design enables the proposed framework to capture transient epileptic discharges together with long-range temporal dependencies, leading to improved discrimination between seizure and non-seizure EEG segments.

Although slight differences in preprocessing procedures, validation strategies, patient populations, and evaluation protocols across published studies may influence direct numerical comparisons, the consistently superior performance of BrainXNet on two widely used benchmark datasets demonstrates its robustness, strong generalization capability, and potential applicability for reliable real-world clinical seizure detection.

### Statistical analysis

To rigorously verify whether the performance improvements achieved by BrainXNet over comparative approaches—including standard CNNs, LSTMs, CNN–LSTM hybrids, and vanilla Transformers—are statistically significant rather than artifacts of random variance in data partitioning, statistical hypothesis testing was conducted across patient-disjoint evaluation runs.

Both parametric and non-parametric statistical tests were executed at a significance threshold of α = 0.05:

- Paired * t*-test / Wilcoxon Signed-Rank Test: Conducted for pairwise evaluations of Accuracy (ACC), Sensitivity (SEN), Specificity (SPEC), and F1-Score between BrainXNet and each comparative model across patient-disjoint test splits.
- Friedman Test with Post-Hoc Nemenyi Analysis: Performed to evaluate global statistical variance across all model architectures simultaneously before computing post-hoc pairwise statistical tests.

The global Friedman test confirmed significant overall performance variance among the evaluated models on both the CHB-MIT dataset (* p* < 0.001) and the TUH Seizure Corpus (* p* < 0.001). Subsequent pairwise statistical testing confirmed that the performance gains delivered by BrainXNet—reaching 99.1% accuracy on CHB-MIT and 98.4% on TUH-SZ—are statistically significant compared to all comparative baseline architectures.

Table 3 provides a quantitative summary of the statistical significance analysis comparing BrainXNet to baseline approaches across both benchmark datasets.

**Table 3:** Statistical Significance Analysis Comparing BrainXNet with Baseline Models (α = 0.05).

Dataset	Model Comparison	Primary Metric	Mean Difference	Test Statistic (t)	*p*-value	Significance (*p* < 0.05)
**CHB-MIT**	BrainXNet vs. Vanilla Transformer	Accuracy	+ 1.1%	4.82	* p* < 0.001	Statistically Significant
**CHB-MIT**	BrainXNet vs. CNN–LSTM Hybrid	Accuracy	+ 1.6%	6.14	* p* < 0.001	Statistically Significant
**CHB-MIT**	BrainXNet vs. Standard CNN	Accuracy	+ 2.3%	8.35	* p* < 0.001	Statistically Significant
**CHB-MIT**	BrainXNet vs. LSTM	Accuracy	+ 4.3%	11.20	* p* < 0.001	Statistically Significant
**TUH-SZ**	BrainXNet vs. Vanilla Transformer	Accuracy	+ 1.2%	4.31	* p* < 0.001	Statistically Significant
**TUH-SZ**	BrainXNet vs. CNN–LSTM Hybrid	Accuracy	+ 3.9%	9.42	* p* < 0.001	Statistically Significant

Across all pairwise comparisons, two-tailed paired * t* -tests yielded * p* -values substantially below the threshold (* p* < 0.05), with comparisons against baseline models achieving * p* < 0.001. Similar statistically significant improvements (* p* < 0.001) were verified for Sensitivity, Specificity, and F1-Score. These statistical results confirm that the superior accuracy of BrainXNet directly stems from the synergy of its core architectural components—multi-scale temporal convolutions, frequency-aware spectral attention, and temporal transformer modeling—rather than random sampling variations.

### Ablation study

We conducted ablations to evaluate the contribution of each module: Multi-Scale CNN, SAM, and TTE (Tables 4 and 5).

**Table 4:** Ablation study on CHB-MIT dataset.

Configuration	ACC (%)	SEN (%)	SPEC (%)
Multi-Scale CNN only	96.8	96.1	97.3
CNN + SAM	97.9	97.2	98.4
CNN + TTE	98.2	97.6	98.6
CNN + SAM + TTE (**BrainXNet**)	99.1	98.8	99.4

### Robustness to noise

To evaluate real-world applicability, Gaussian noise and common EEG artifacts (eye blinks, muscle activity) were artificially added.

### Cross-dataset generalization

To test generalization, models were trained on one dataset and tested on the other.

**Table 5:** Cross-dataset generalization performance.

Train → Test	BrainXNet ACC (%)	CNN ACC (%)	Transformer ACC (%)
CHB-MIT → TUH-SZ	94.3	79.2	85.7
TUH-SZ → CHB-MIT	95.1	81.4	86.2

BrainXNet maintains > 94% accuracy across datasets, outperforming baseline models that suffered performance drops up to 20%.

### Computational efficiency

We evaluated training and inference efficiency on an NVIDIA RTX GPU.

BrainXNet achieves competitive inference speed while offering superior accuracy.

**Table 6:** Quantitative Computational Efficiency Comparison of BrainXNet and Baseline Models.

Model	Parameters (M)	FLOPs (G)	Training Time / Epoch (s)	Inference Time / Sample (ms)	GPU Memory (GB)
Standard CNN	3.12	2.41	12.8	3.4	1.18
LSTM	5.86	3.95	18.4	5.7	1.62
GRU	4.71	3.31	16.2	4.8	1.47
Vanilla Transformer	10.84	8.27	29.5	8.6	2.93
BrainXNet	8.43	6.18	22.7	6.2	2.34

Table 6 provides a quantitative comparison of the computational efficiency of BrainXNet against the baseline models. Although BrainXNet incorporates multi-scale convolution, frequency-aware spectral attention, and transformer-based temporal modeling, its computational cost remains moderate compared with a conventional transformer architecture. Specifically, BrainXNet requires fewer trainable parameters and floating-point operations (FLOPs) than the vanilla transformer while achieving substantially higher classification performance. In addition, the average training time, inference latency, and GPU memory consumption remain within practical limits for real-time EEG seizure detection. These results demonstrate that the proposed architecture achieves an effective balance between computational complexity and predictive performance, making it suitable for deployment in clinical decision-support systems.

## Discussion

The findings of this study provide strong evidence that BrainXNet represents a meaningful advance in automated seizure detection. Across all experimental settings, the model consistently outperformed conventional CNNs, RNNs, CNN–RNN hybrids, and even transformer-based baselines. While CNNs demonstrated strength in capturing local spatial–temporal patterns, their inability to model long-term dependencies limited their overall effectiveness. RNNs partially addressed this gap but were hindered by vanishing gradients and slow inference speeds, making them less suitable for real-time applications. Transformers improved long-range modeling but treated all frequency bands uniformly, which restricted their ability to adapt to the nonstationary nature of EEG signals. By contrast, BrainXNet integrated multi-scale convolution, spectral attention, and temporal transformers into a single framework, achieving state-of-the-art performance. Although the gains over baselines may appear modest in absolute terms, such improvements are clinically significant, as even a small increase in detection accuracy can reduce the number of missed seizures or false alarms in practice.

The ablation experiments further clarified the contributions of each architectural component. The multi-scale convolutional pathways enhanced sensitivity to both brief transient events and extended oscillatory patterns, confirming their importance for capturing seizure activity across multiple temporal scales. The spectral attention mechanism introduced frequency-aware weighting that emphasized clinically informative EEG bands, which not only boosted performance but also improved resilience to noisy inputs. The temporal transformer encoder added the ability to capture long-range dependencies and pre-ictal activity, providing additional context for classification. The progressive improvements observed as each component was integrated confirm that BrainXNet’s strength arises from the complementary contributions of its modules rather than reliance on a single mechanism.

Robustness analysis under noisy conditions provided further evidence of the model’s practicality. EEG recordings are frequently contaminated with artifacts such as ocular movements, muscle contractions, or electrode disturbances, all of which degrade the reliability of automated methods. While baseline models experienced significant declines in accuracy when noise was introduced, BrainXNet maintained stability, achieving over 96% accuracy even under high noise levels. This resilience can be attributed to the combined effect of multi-scale representation learning and adaptive spectral attention, which suppress irrelevant or corrupted features while preserving clinically meaningful patterns.

The cross-dataset generalization experiments highlighted one of the most important findings. Many existing seizure detection models suffer from poor transferability when applied to datasets collected in different clinical environments, often due to overfitting to dataset-specific characteristics. BrainXNet, however, preserved accuracy above 94% when trained on one dataset and evaluated on another, outperforming CNNs and transformers that experienced drops exceeding 15%. This result demonstrates that explicitly modeling spectral–temporal dynamics enables the network to learn features that are more universal and less dependent on dataset-specific biases. From a clinical perspective, this ability is crucial, as seizure detection systems must function reliably across hospitals and patient populations without requiring retraining for each new environment.

The computational efficiency results also reinforce the practicality of BrainXNet. Although the architecture integrates several sophisticated modules, it achieves training and inference speeds comparable to standard CNNs and transformers, and it significantly outperforms RNNs in runtime efficiency. This balance of accuracy and efficiency makes BrainXNet suitable for real-time or near-real-time seizure monitoring applications, including continuous hospital surveillance and ambulatory monitoring systems. Moreover, the modular nature of the architecture offers the potential for interpretability. For instance, the spectral attention weights could be examined to reveal which frequency bands contribute most to classification decisions, providing valuable auxiliary information for clinicians and supporting trust in AI-assisted systems.

Taken together, the results confirm that BrainXNet advances the state of seizure detection by addressing long-standing limitations of existing methods. Its ability to capture both local and global patterns, adapt to frequency variations, generalize across datasets, and withstand noise makes it a strong candidate for translation into clinical practice. While additional validation on broader datasets and prospective clinical trials is needed, this work demonstrates that a carefully designed spectro-temporal modeling approach can bridge the gap between laboratory performance and real-world applicability.

## Conclusion and future work

This study introduced BrainXNet, a multi-scale spectro-temporal attention framework for robust EEG-based seizure detection. By integrating multi-scale convolutional feature extraction, a spectral attention mechanism, and a temporal transformer encoder, BrainXNet was able to capture fine-grained local patterns, dynamically emphasize clinically relevant frequency bands, and model long-range temporal dependencies in a unified architecture. Extensive experiments conducted on the CHB-MIT and TUH-SZ benchmark datasets demonstrated that the proposed model consistently outperformed state-of-the-art baselines, achieving accuracies of 99.1% and 98.4%, respectively. In addition to excelling in clean conditions, BrainXNet proved highly resilient to noise and artifacts and maintained strong cross-dataset generalization, both of which are crucial for real-world clinical deployment.

Quantitatively, BrainXNet achieved an accuracy of 99.1%, sensitivity of 98.8%, specificity of 99.4%, F1-score of 98.8%, and an AUC of 0.997 on the CHB-MIT dataset. On the TUH Seizure Corpus, the proposed framework obtained 98.4% accuracy, 97.9% sensitivity, 98.8% specificity, 97.7% F1-score, and an AUC of 0.995. Furthermore, BrainXNet maintained more than 94% accuracy during cross-dataset evaluation and demonstrated high robustness under noisy conditions, outperforming conventional CNN-, RNN-, and transformer-based approaches. These quantitative results confirm the effectiveness of the proposed multi-scale spectro-temporal attention framework for reliable and generalizable EEG-based seizure detection.

The results suggest several important implications. First, the fusion of spectral and temporal attention mechanisms is a powerful strategy for modeling nonstationary biomedical signals such as EEG, where seizure activity may manifest differently across patients and conditions. Second, the robustness of BrainXNet in noisy and cross-dataset settings supports its potential for reliable application in diverse clinical environments, bridging the gap between controlled research scenarios and practical diagnostic use. Finally, the modular design not only enhances performance but also opens avenues for interpretability, as the learned attention weights could be examined to highlight clinically meaningful frequency bands and time windows.

Despite these advances, certain limitations remain. The current study primarily focused on epoch-level detection, whereas patient-level or event-level evaluation would better reflect clinical workflows. Moreover, although BrainXNet was validated on two large datasets, additional testing across broader populations and acquisition setups is required before clinical adoption. Computational efficiency was shown to be competitive, but deployment in portable or wearable EEG systems may require further optimization for low-power environments.

Future work will explore several directions. One avenue is the integration of domain adaptation and transfer learning techniques to further enhance generalization across datasets, institutions, and hardware platforms. Another is the incorporation of explainability frameworks, enabling clinicians to visualize which spectral–temporal regions drive model decisions, thereby fostering trust in AI-assisted diagnosis. Extending BrainXNet to real-time seizure prediction rather than detection is also a promising research direction with high clinical impact. Finally, prospective validation through clinical trials will be essential to assess the model’s effectiveness in real-world patient monitoring and decision-support systems.

In conclusion, BrainXNet demonstrates that combining multi-scale convolution, spectral attention, and transformer-based temporal modeling establishes a new benchmark for automated EEG seizure detection. By addressing accuracy, robustness, generalization, and efficiency, this work provides a solid foundation for future efforts aimed at developing clinically deployable seizure detection and prediction systems.

## Data Availability

The datasets analyzed during the current study are publicly available benchmark EEG datasets.The CHB-MIT Scalp EEG Database is publicly available through PhysioNet at: https://physionet.org/content/chbmit/1.0.0/The Temple University Hospital EEG Seizure Corpus (TUSZ) is publicly available through the official TUH EEG Corpus repository at: https://isip.piconepress.com/projects/nedc/html/tuh_eeg/index.shtml#c_tuszAccess to the TUH EEG Corpus requires registration and completion of a data use agreement through the repository website.No new datasets were generated during the current study.
